# Anti-Mullerian Hormone As A Predictor Of Live Birth For Patients With Unexplained Recurrent Pregnancy Loss

**DOI:** 10.5935/1518-0557.20260002

**Published:** 2026

**Authors:** Edward R. McClellan, Yael Eliner, Hillary Pearson, Timothy J. Rafael, Ji Yoon Lee, Randi Goldman, Mary Rausch

**Affiliations:** 1 Northwell, New Hyde Park, NY; Department of Obstetrics and Gynecology, Division of Human Reproduction, Manhasset, NY; USA, Zucker School of Medicine, Uniondale, NY, USA; 2 AMEDD Student Detachment, 187th Medical Battalion, 32nd Medical Brigade, Fort Sam Houston, TX, USA; 3 Northwell, New Hyde Park, NY; Lenox Hospital Obstetrics and Gynecology Residency Program, New York, NY, USA; 4 Northwell, New Hyde Park, NY; Department of Obstetrics and Gynecology, Division of Maternal-Fetal Medicine, Manhasset, NY; Zucker School of Medicine, Uniondale, NY, USA; 5 Northwell, New Hyde Park, NY; Department of Biostatistics, Feinstein Institutes for Medical Research, Northwell Health, Manhasset, NY, USA

**Keywords:** AMH, RPL, euploid FET, unexplained recurrent pregnancy loss, URPL

## Abstract

**Objective:**

To determine whether anti-mullerian hormone (AMH) levels were associated with live birth and miscarriage rates following the first euploid frozen embryo transfer (FET) cycle for patients with unexplained recurrent pregnancy loss (URPL).

**Methods:**

This retrospective cohort study included all patients (n=63) at an academic medical center in New York undergoing their first euploid FET with a sole diagnosis of URPL between October 2019 and October 2023. URPL was defined as a history of two or more spontaneous abortions (SAB) after a pregnancy (defined as a positive home pregnancy test or serum hCG > 2mIU/mL) and before 20 weeks of gestation without a known cause. Patient demographic data was obtained, and the primary outcomes of live birth and miscarriage were assessed while controlling for relevant clinical variables (obesity, prior number of spontaneous abortions, and aneuploidy). Significance was set at *p*<0.05.

**Results:**

Lower AMH (<1 ng/mL) was associated with having fewer total blastocysts from prior IVF cycles (*p*=0.02). Patients with lower AMH also had fewer euploid blastocysts from prior IVF cycles, although this difference was not statistically significant (*p*=0.08). There were no significant differences based on AMH, and the overall miscarriage rate was 21.3%. Adjusting for obesity and prior number of SABs, AMH was not associated with live birth (aOR 1.12 (95% CI 0.93-1.35), *p*=0.23) or miscarriage (aOR 0.88 (95% CI 0.64-1.19), *p*=0.40).

**Conclusions:**

These results suggest that once a euploid embryo is created, AMH is not associated with subsequent transfer outcomes among women with URPL.

## INTRODUCTION

AMH is not associated with reduced fecundability among women without a history of infertility (Steiner *et al.*, 2017), nor is AMH associated with fecundability among women with a history of one or two prior spontaneous abortions (Zarek *et al.*, 2015). However, lower AMH is a possible risk factor for miscarriage in naturally conceived pregnancies (Lyttle Schumacher *et al.*, 2018) and has also been associated with lower implantation and clinical pregnancy rates among women undergoing In-Vitro Fertilization (IVF) cycles (Tal *et al.*, 2015).

While low anti-mullerian hormone (AMH) levels have been associated with recurrent pregnancy loss (RPL) among women with RPL (Bunnewell *et al.*, 2020), this association has been confounded by age and unknown ploidy status (Murugappan *et al.*, 2019; Bunnewell *et al.*, 2020). It has been hypothesized that the reported association between AMH and RPL may be due to AMH’s role as a putative marker of oocyte quality, perhaps independent of aneuploidy (Murugappan *et al.*, 2019; Bunnewell *et al.*, 2020); however, others argue that AMH is a marker only of quantity, not quality, of oocytes (Tal & Seifer, 2017). Because prior studies have not assessed the association between AMH and RPL among patients using PGT-A tested embryos, the role of AMH as a prognosticator of pregnancy outcomes has thus far been confounded by unknown ploidy status, and the relevance of AMH for women with RPL transferring PGT-A tested euploid embryos remains unknown (Murugappan *et al.*, 2019).

Despite insufficient evidence that PGT-A increases live birth rates for patients with unexplained recurrent pregnancy loss (URPL), this treatment is pursued by some with URPL because aneuploidy is the major cause of all miscarriages (Practice Committee of the American Society for Reproductive Medicine, 2012). Prior research has noted similar IVF and pregnancy outcomes among patients with RPL compared to patients with other infertility diagnoses (Kornfield *et al.*, 2022). This previous study controlled for AMH; however, it reported neither the outcomes of the first euploid frozen embryo transfer (FET) in their RPL cohort nor their pregnancy rates per transfer (Kornfield *et al.*, 2022).

Our study aimed to determine whether AMH levels were associated with live birth and miscarriage following the first euploid frozen embryo transfer (FET) cycle for patients with URPL.

## MATERIAL AND METHODS

This is a retrospective cohort study of all patients (n=63) at an academic institution undergoing their first autologous, euploid FET with a sole diagnosis of URPL between October 2019 and October 2023. URPL was defined as a history of two or more spontaneous abortions (SAB) after a pregnancy (defined as a positive home pregnancy test or serum hCG > 2mIU/mL) and before 20 weeks of gestation without a known cause (Practice Committee of the American Society for Reproductive Medicine, 2012). A complete RPL workup included completing all of the following: parental karyotypic evaluation, serum screening for antiphospholipid antibody syndrome, either sonohysterogram or hysterosalpingogram, and serum screening for thyroid and prolactin disorders (Practice Committee of the American Society for Reproductive Medicine, 2012). Patients were excluded from analysis if a known etiology for their losses was present or if they did not have a completed evaluation performed before their first euploid FET.

This study was approved by the Feinstein Institute for Medical Research Institutional Review Board (22-1015). Fisher’s Exact test and Kruskal-Wallis test were performed with *p*<0.05 considered statistically significant. For significant Kruskal-Wallis test results, pairwise Wilcoxon rank-sum tests were conducted with Bonferroni correction for multiple comparisons. Multivariate logistic regression models were examined, and adjusted odds ratios were calculated, controlling for two clinically relevant variables (obesity and prior number of spontaneous abortions), to maintain an acceptable events-per-variable ratio. Aneuploidy was inherently controlled for by the study design.

## RESULTS

Table 1 reports cycle characteristics with additional information provided in Supplementary Table 1. A lower AMH level was associated with having fewer total blastocysts from prior IVF cycles (*p*=0.02). Patients with lower AMH also had fewer euploid blastocysts from prior IVF cycles, although this difference was not statistically significant (*p*=0.08). Pregnancy outcomes are reported in Table 2. There were no significant differences based on AMH, and the overall miscarriage rate was 21.3%. Adjusting for obesity and prior number of spontaneous abortions, AMH was not associated with live birth (aOR 1.12 (95% CI 0.93-1.35), *p*=0.23) or miscarriage (aOR 0.88 (95% CI 0.64-1.19), *p*=0.40).

**Table 1:** Cycle Characteristics.

Characteristics median (IQR)	All Patients n=63	AMH			p-value
		<1 ng/mL n=6	1-3.5 ng/mL n=32	>3.5 ng/mL n=25	
**Age**	35.0 (33.0, 38.0)	38.8 (35.0, 40.0)	35.0 (33.0, 37.5)	35.0 (32.0, 38.0)	0.28
**BMI**	26.0 (23.2, 33.2)	28.9 (26.8, 30.1)	25.7 (22.8, 33.4)	25.5 (23.6, 30.7)	0.92
**Prior Spontaneous Abortions**	2.0 (2.0, 3.0)	2.0 (2.0, 3.0)	2.0 (2.0, 3.0)	2.0 (2.0, 3.0)	0.96
**Prior Therapeutic Abortions**	0 (0, 0)	0 (0, 0)	0 (0, 0)	0 (0, 0)	0.96
**Prior Ectopic Pregnancies**	0 (0, 0)	0 (0, 0)	0 (0, 0)	0 (0, 0)	none
**Prior Live Births**	0 (0, 1.0)	0.5 (0, 1.0)	0 (0, 1.0)	0 (0, 1.0)	0.90
**Number IVF Cycles Completed**	1.0 (1.0, 1.0)	1.0 (1.0, 1.0)	1.0 (1.0, 1.5)	1.0 (1.0, 1.0)	0.77
**Number IVF Cycles Canceled**	0 (0, 0)	0 (0, 0)	0 (0, 0)	0 (0, 0)	0.46
Total Blastocysts^*^	8.0 (4.0, 10.0)	4.0 (3.0, 5.0)	7.0 (4.0, 10.0)	9.0 (7.0, 13.0)	0.02
**Euploid Blastocysts**	4.0 (2.0, 5.0)	3.0 (1.0, 4.0)	4.0 (3.0, 5.0)	9.0 (7.0, 13.0)	0.08

IQR = Interquartile Range

Kruskal-Wallis test

* After obtaining significant differences in the total number of blastocysts with the Kruskal-Wallis test, pairwise Wilcoxon rank-sum tests with Bonferroni correction were performed (significance level: 0.05/3 = 0.0167). A significant difference was observed between patients with AMH<1 ng/mL and AMH > 3.5 ng/mL (*p*=0.007). However, no significant differences were observed between patients with AMH<1 ng/mL and AMH 1-3.5 ng/mL (*p*=0.03) or between patients with AMH 1-3.5 ng/mL and AMH > 3.5 ng/mL (*p*=0.16).

**Table 2:** Transfer Outcomes.

Results	All Patients n=63	AMH <1 ng/mL n=6	AMH 1-3.5 ng/mL n=32	AMH> 3.5 ng/mL n=25	pvalue
**Pregnancy n (%)**	48 (76.2)	5 (83.3)	23 (71.9)	20 (80.0)	0.83
**Clinical Pregnancy n (%)**	39 (61.9)	3 (50.0)	20 (62.5)	16 (64.0)	0.86
**Live Birth n (%)**	37 (58.7)	3 (50.0)	18 (56.3)	16 (64.0)	0.75
Miscarriage^*^^ n (%)	10 (21.3)	2 (40.0)	5 (21.7)	3 (15.8)	0.46

Fisher's Exact Test

* The miscarriage rate was calculated for 47 patients with a positive pregnancy test.

^One patient with an AMH > 3.5 had an ectopic pregnancy

## DISCUSSION

This is the first known study to assess whether AMH levels are associated with live birth and miscarriage following the first euploid FET cycle for patients with URPL. A prior systematic review and meta-analysis showed that low AMH is associated with subsequent pregnancy loss among patients with RPL; however, the authors were unable to adjust for age (Bunnewell *et al.*, 2020). Age is known to affect both AMH and euploid rates, and there is evidence that AMH may be associated with euploid rates even when adjusting for age (Jaswa *et al.*, 2021). Our results showed no association between AMH and either live birth or miscarriage rates among patients with URPL. Lower AMH was associated with having fewer total blastocysts although the number of euploid blastocysts from prior IVF cycles was not significantly different among groups.

These results of no association between AMH and subsequent FET outcomes suggest that prior associations between AMH and RPL noted by some authors may have been confounded by age and aneuploidy, because in our study, once a euploid embryo was created, AMH was not associated with subsequent transfer outcomes among women with URPL.

The study’s major limitations include our small sample size, the small number of patients with an AMH<1ng/mL, and our retrospective study design. The study’s main strengths are the strict inclusion criteria utilized for defining URPL and novelty. Additionally, this manuscript can inform future research into this important, understudied area of reproductive medicine. Based on the estimates of this study, a sample size of 241 patients in each of the three AMH level groups would be needed to achieve 80% power using a Chi-square test with a significance level of *p*<0.05.

## CONCLUSION

These results suggest that AMH is not associated with live birth or miscarriage rates among patients with URPL undergoing their first euploid FET cycle. Future research is needed to determine if this finding remains true for patients with URPL and an AMH<1ng/mL.

## Figures and Tables

**Supplementary Table 1:** 

Study Population	All Patients	AMH			*p*-value
		<1 ng/mL	1-3.5 ng/mL	>3.5 ng/mL	
	n=63	n=6	n=32	n=25	
	n (%)	n (%)	n (%)	n (%)	
Race Asian Black White Other Declined/Unknown	6 (9.5) 6 (9.5) 32 (50.8) 11 (17.5) 8 (12.7)	0 (0) 1 (16.7) 3 (50.0) 0 (0) 2 (33.3)	4 (12.5) 2 (6.3) 15 (46.9) 6 (18.8) 4 (12.5)	2 (8.0) 3 (12.0) 14 (56.0) 5 (20.0) 1 (4.0)	0.54
**Ethnicity** **Hispanic** **Non-Hispanic** **Declined/Unknown**	3 (4.8) 53 (84.1) 7 (11.1)	0 (0) 4 (66.7) 2 (33.3)	1 (3.1) 27 (84.4) 4 (12.5)	2 (8.0) 22 (88.0) 1 (4.0)	0.24
**Cycle Type** **Natural Cycle** **Programmed Cycle**	16 (25.4) 47 (74.6)	3 (50.0) 3 (50.0)	10 (31.3) 22 (68.8)	3 (12.0) 22 (88.0)	0.08

Fisher's Exact Test.
